# Identification of genes for engineering the male germline of *Aedes aegypti* and *Ceratitis capitata*

**DOI:** 10.1186/s12864-016-3280-3

**Published:** 2016-11-21

**Authors:** Elizabeth R. Sutton, Yachuan Yu, Sebastian M. Shimeld, Helen White-Cooper, and Luke Alphey

**Affiliations:** 1 Department of Zoology, University of Oxford, Oxford, OX1 3PS UK; 2 Oxitec Ltd, Milton Park, Abingdon, OX14 4RX UK; 3 School of Biosciences, Cardiff University, Cardiff, CF10 3AX UK; 4 The Pirbright Institute, Pirbright, GU24 0NF UK; 5 Present address: Sistemic, West of Scotland Science Park, Glasgow, G20 0SP UK; 6 Present address: The Beatson Institute for Cancer Research, CRUK, Glasgow, G61 1BD UK

**Keywords:** Synthetic biology, Pest insect, Male germline, RNA-seq, *Aedes aegypti*, *Ceratitis capitata*

## Abstract

**Background:**

Synthetic biology approaches are promising new strategies for control of pest insects that transmit disease and cause agricultural damage. These strategies require characterised modular components that can direct appropriate expression of effector sequences, with components conserved across species being particularly useful. The goal of this study was to identify genes from which new potential components could be derived for manipulation of the male germline in two major pest species, the mosquito *Aedes aegypti* and the tephritid fruit fly *Ceratitis capitata*.

**Results:**

Using RNA-seq data from staged testis samples, we identified several candidate genes with testis-specific expression and suitable expression timing for use of their regulatory regions in synthetic control constructs. We also developed a novel computational pipeline to identify candidate genes with testis-specific splicing from this data; use of alternative splicing is another method for restricting expression in synthetic systems. Some of the genes identified display testis-specific expression or splicing that is conserved across species; these are particularly promising candidates for construct development.

**Conclusions:**

In this study we have identified a set of genes with testis-specific expression or splicing. In addition to their interest from a basic biology perspective, these findings provide a basis from which to develop synthetic systems to control important pest insects via manipulation of the male germline.

**Electronic supplementary material:**

The online version of this article (doi:10.1186/s12864-016-3280-3) contains supplementary material, which is available to authorized users.

## Background

Insects pose large problems for human health and agriculture; several major global diseases are transmitted by insect vectors, and huge losses in food production occur due to insect pests.

Current strategies for insect control have a number of disadvantages, such as effects on non-target species and development of resistance to insecticides [1]. Alternative synthetic biology approaches are being developed in which the control agent is a modified version of the pest insect itself. These modified insects carry a genetic system that results in the death of some or all of their descendants, so that when released modified insects mate with wild counterparts, population suppression occurs. Such strategies require characterised modular components that can direct appropriate expression of effector sequences – protein-coding sequences or functional RNAs, for example. Conserved components that can be used across multiple species are particularly useful. However, for many applications there are few if any such components available. The goal of this study was to identify genes that could provide potential components for manipulation of the male germline in two major pest species, the mosquito *Aedes aegypti* (L.) and the tephritid fruit fly *Ceratitis capitata* (Wiedemann).

These species were selected because of their importance to public health and agriculture, respectively. *Ae. aegypti* vectors a number of viral diseases including dengue fever [2], the most prevalent mosquito-borne viral disease, with an estimated 390 million infections per year [3]. There is no specific therapeutic or prophylactic treatment, and no licensed vaccine, meaning vector control is currently the only option for prevention. *C. capitata* (Mediterranean fruit fly, medfly), is a widespread, economically important agricultural insect pest, affecting over 250 types of crop [4]. The choice of these two distantly related species also allowed us to search for genes that may be conserved across multiple species.

Genetic insect control systems require expression of the effector transgene in a particular tissue and/or at a particular developmental stage, and usually require that the transgene not be expressed elsewhere or at another time. While some genetic control methods and strains have been successfully developed based on ubiquitous or targeted expression in somatic tissues [5–13], for several potential strategies, germline-specific transgene expression is required, male germline-specific expression being of particular interest. These include sex-ratio distortion systems, which involve the release of males carrying a transgene whose product selectively destroys sperm that would result in female offspring. The resultant skewing of the sex ratio towards males would lead to population suppression [14–17]. Other approaches would eliminate sperm production [15], or lead to the death of embryos fertilised by sperm from modified males [17].

Though many types of regulatory element might in principle be used, in practice expression is usually controlled by the choice of promoter. Alternative splicing cassettes may also be used, either with a non-specific or specific promoter. For example, sex-specific alternative splicing has been used to achieve female-specific expression in *C. capitata* [8], olive fly [9], pink bollworm and diamondback moth [10], and to add additional specificity to an already sex-biased promoter in *Ae. aegypti* [11], *Ae. albopictus* [12] and *Anopheles stephensi* [13]. Analogous components to drive germline-specific expression, particularly in males, would be useful for the applications described above.

**Fig. 1:**
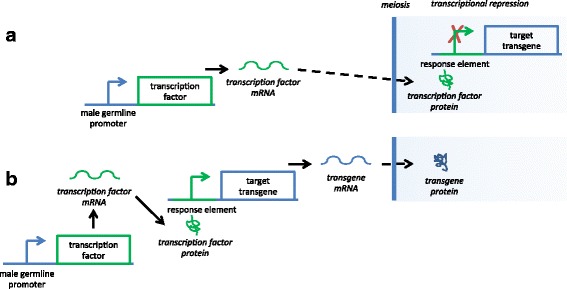
Importance of pre-meiotic protein expression in bipartite synthetic genetic systems. If transcription is repressed from meiosis onwards, post-meiotic translation of the transcription factor in a bipartite expression system is not adequate for expression of the target transgene (**a**). Expression of the transgene requires translation of the transcription factor before meiosis such that the target transgene is transcribed before transcriptional repression at meiosis (**b**)

Several insect genes with testis-specific expression have been identified, often first in *Drosophila melanogaster* (Meigen), for example *β2-tubulin* [18]. Homologues of *β2-tubulin* have been identified and the promoters found to drive testis-specific expression in other species, including *Anopheles gambiae* [19], *Ae. aegypti* [20] and *C. capitata* [21]*.* However, studies on *D. melanogaster* suggest that expression timing in male germline cells must be taken into consideration. In *D. melanogaster*, transcription is repressed with the onset of the meiotic divisions [22, 23]. Barring a few exceptions [24–26], genes whose protein product is required after this transcriptional repression are transcribed in primary spermatocytes, before the meiotic divisions; the transcripts are then stored and translated as required [27]. Though not studied in detail for other insects, the major changes to chromatin structure at meiosis and subsequently suggest this may be a general phenomenon. Testis-specific bipartite synthetic genetic systems involving transcription factors (e.g. GAL4 or tTA, both widely used in insect synthetic biology [28, 29]) would therefore require regulatory regions (promoters and/or UTRs) that drive pre-meiotic protein expression, otherwise the transcription factor would not be translated early enough to drive transcription of its target (Fig. 1). While promoters may control tissue specificity, it is likely that timing of translation is controlled by UTRs (though in prokaryotes translation has been shown to be affected by promoter sequences [30]), so identification of both promoters and UTRs is likely to be important.

High-throughput transcriptional profiling [31] and subtractive hybridisation [32] studies have recently yielded several potential testis-specific transcripts in *Ae. aegypti*. However, to our knowledge, no studies have been performed with sufficient time resolution to determine the activity of regulatory regions at different stages of spermatogenesis. Information on insect testis-specific splicing is even more sparse; testis-specific splice forms of the genes *achi* and *vis* have been discovered in *D. melanogaster* [33], but no testis-specific splice forms have been identified, to our knowledge, in *Ae. aegypti, C. capitata* or any other pest insect.

In this study we performed RNA-seq on staged testis samples from *Ae. aegypti* and *C. capitata*, to identify genes with testis-specific expression peaking early in spermatogenesis, whose regulatory regions are therefore candidates for driving pre-meiotic protein expression. We also developed a novel computational pipeline to identify testis-specific splice forms that could potentially provide additional tools for germline-specific genetic systems. By comparing results from the two species, we have attempted to identify conserved components that may function in constructs across multiple species. In addition to their use in applied synthetic biology, these elements are also interesting from a basic biology perspective.

## Results

### RNA sequencing and read alignment

RNA sequencing was performed on eight samples, two *Ae. aegypti* and four *C. capitata* dissected testis samples representing different spermatogenesis stages, an *Ae. aegypti* gonadectomised male sample, and a *C. capitata* ovary sample. The two *Ae. aegypti* testis samples were generated by bisecting testes and will be referred to as “early” and “late”*.* The four *C. capitata* testis samples constituted early spermatocytes, late spermatocytes, round spermatids and elongated spermatids, respectively. Sequencing was performed using the Illumina Genome Analyzer II platform with single reads of 73 nucleotides. In total 255,090,176 reads were generated for the eight samples, corresponding to 18.6 Gb of data, with 89.1% of *Ae. aegypti* reads and 89.8% of *C. capitata* reads aligning to the corresponding genome (see Additional file 1 for more details).

Data from *C. capitata* female and *Ae. aegypti* female and ovary samples from other experiments were downloaded from the Sequence Read Archive (SRA) [34]. The *Ae. aegypti* female sample was gonadectomised; an ovary sample was therefore used in addition so that data from all female tissues were present. The *C. capitata* female sample was not gonadectomised, but an ovary sample was still sequenced and included in the analysis, as many genes expressed in the testis could potentially also be expressed in the ovary, and their detection may be impeded by the large amount of other tissue in a whole female sample. The *Ae. aegypti* ovary and female samples were from recently fed females (~24 h post-blood meal), as these will include transcripts expressed during oogenesis, thus enabling elimination of genes expressed in both male and female gametogenesis. The number of reads in these samples and the proportion aligning to the corresponding genome are shown in Additional file 1.

### Identification of candidate testis-specifically expressed genes

Candidate testis-specifically expressed genes were identified from the total set of predicted genes by running a custom Python script on the output of the standard TopHat-Cufflinks-Cuffdiff RNA-seq analysis pipeline, and applying various filtering steps (described below) to maximise sensitivity whilst removing unsuitable genes and minimising false positives.

An expression level of 10 FPKM (fragments per kilobase of exon per million fragments mapped) in the early sample for *Ae. aegypti* and the early spermatocytes sample for *C. capitata* was chosen as a threshold for candidates. A threshold was set as predicted genes with low expression are more likely to be false positives, and also regulatory elements associated with relatively strong expression are desired for use in synthetic constructs; 10 FPKM is the boundary between low and moderate expression for *D. melanogaster* RNA-seq data on FlyBase [35]. The threshold for expression in samples other than testis (gonadectomised male, ovary and female) was not set at zero, to allow for some noise in the data, but rather at 1 FPKM, based on quantification of the known testis-specifically expressed genes *can*, *comr*, *nht* and *Taf12L* in *D. melanogaster* (data not shown).

Many potential candidates appeared to be short non-coding RNAs. Quantification of short non-coding RNAs is likely to be inaccurate in a protocol using polyA selection. Therefore the only genes taken forward for further analysis were those that either coincided with a locus already annotated as a protein-coding gene, or novel predicted genes that were over 1 Kb in length.

After application of the filtering steps above, predicted testis-specifically expressed genes with higher expression in early spermatogenesis than in late spermatogenesis were identified. For *Ae. aegypti*, 57 candidate early genes were identified, out of a total of 388 predicted testis-specifically expressed genes with expression above 10 FPKM in the early sample. For *C. capitata*, 68 candidate early genes were identified, out of a total of 667 predicted testis-specifically expressed genes with expression above 10 FPKM in early spermatocytes.

For each species, the top ten candidates in order of expression level in the earliest testis sample were taken forward for experimental testing. Genes encoding proteins associated with transposable elements were excluded, as there are likely to be multiple copies of these in the genome, and it would be difficult to design PCR primers that would target only one. For *Ae. aegypti*, one additional candidate was also taken forward, as a homologue of the gene was identified as a candidate in *C. capitata*; candidates that are conserved between species may simplify construct generation in different species. Lists of the candidate genes tested, and the annotated loci that they correspond to, if any, can be seen in Additional file 2.

### Experimental testing of candidate testis-specifically expressed genes

#### RT-PCR

**Fig. 2:**
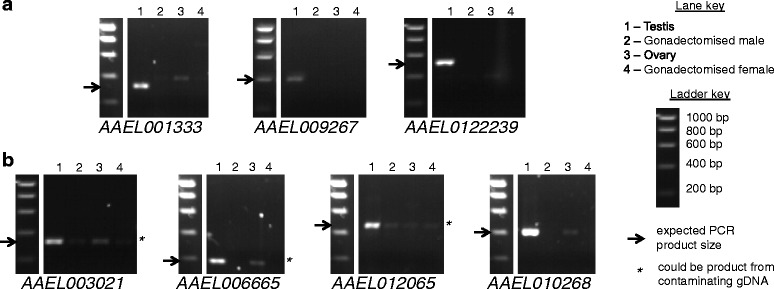
Gels showing PCR results for *Ae. aegypti* expression candidates. **a** Candidates for which no band of the expected size for the testis sample could be seen in non-testis samples. **b** Candidates for which a band of the expected size for the testis sample could be seen in a non-testis sample, but it was faint and in the cases indicated by asterisks, could have resulted from contaminating gDNA. Expected PCR product sizes are indicated with arrows. In some cases bands of other sizes are of the expected size for products amplified from contaminating gDNA. Other bands of unexpected sizes may represent isoforms that were not predicted, or non-specific amplification

**Fig. 3:**
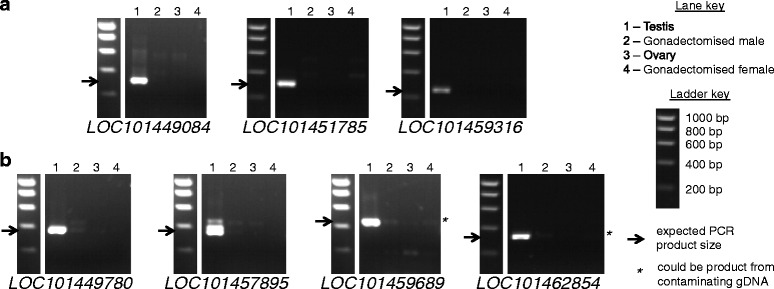
Gels showing PCR results for *C. capitata* expression candidates. Presented as for Fig. 2

Reverse transcriptase PCR (RT-PCR) for the selected candidates was performed on total RNA derived from testis, gonadectomised male, ovary and gonadectomised female samples, to confirm that the candidates were testis-specifically expressed in adults. For some candidates, the RT-PCR results suggested that there was also expression of the gene in other tissues, mostly ovary and one candidate failed to produce a positive result in the testis sample. However, the results supported the prediction of testis-specific expression for several candidates (Figs. 2 and 3), discussed below.

Three candidate *Ae. aegypti* genes (Fig. 2a), corresponding to the annotated loci *AAEL001333*, *AAEL009267* and *AAEL0122239* and three candidate *C. capitata* genes (Fig. 3a), corresponding to the annotated loci *LOC101449084*, *LOC101451785* and *LOC101459316*, displayed the expected outcome of RT-PCR amplification from testis and no amplification from other samples, and were taken forward for further testing. Four additional candidate *Ae. aegypti* genes (Fig. 2b), corresponding to the annotated loci *AAEL003021*, *AAEL006665*, *AAEL010265* and *AAEL010268* and four additional candidate *C. capitata* genes (Fig. 3b), corresponding to the annotated loci *LOC101449780*, *LOC101457895*, *LOC101459689* and *LOC101462854*, were also taken forward despite some amplification in non-testis samples. In these cases the quantity of product from the non-testis samples was low, and in some cases the product could have resulted from amplification of contaminating gDNA.

#### qRT-PCR

**Fig. 4:**
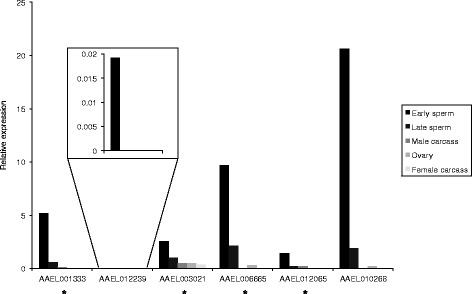
Relative expression levels in different tissues for *Ae. aegypti* expression candidates, determined using qRT-PCR. Results for *AAEL012239* are shown inset, as the expression level for this gene was too low to view at the same scale as for the other genes. * Primers could also have amplified from gDNA, so apparent low expression in non-testis tissues could be a result of gDNA contamination

**Fig. 5:**
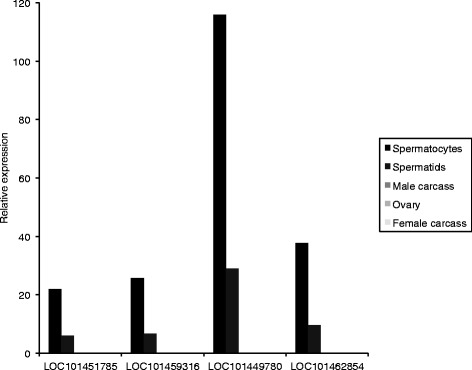
Relative expression levels in different tissues for *C. capitata* expression candidates, determined using qRT-PCR

Quantitative RT-PCR (qRT-PCR) for the candidate genes taken forward for further testing was performed on staged testis samples (early and late samples for *Ae. aegypti*, spermatocytes and spermatids samples for *C. capitata*), to confirm that the candidate genes displayed the desired expression pattern of higher expression early in spermatogenesis (Figs. 4 and 5). Gonadectomised male, ovary and gonadectomised female samples were also used in the qRT-PCR to quantify the level of expression in these tissues, if any. Candidates with a low level of non-testis expression may still be usable for restricting expression to the testis, particularly in combination with other strategies, such as use of testis-specific splicing.

The timing was confirmed for all *Ae. aegypti* candidates (Fig. 4) except *AAEL009267*, for which the qRT-PCR failed, and for four of the *C. capitata* candidates (Fig. 5). For the other three *C. capitata* candidates, *LOC101449084*, *LOC101457895* and *LOC101459689*, no expression was detected in spermatocytes. The results for all the *Ae. aegypti* candidates except *AAEL012239* suggested some expression in non-testis tissues, but this was at a low level compared to that in testis, and in four of the five cases amplification could have resulted from contaminating gDNA. For all the *C. capitata* candidates, no expression was detected in non-testis samples.

### Identification of candidate testis-specifically spliced genes

Similarly to the candidate testis-specifically expressed genes, analysis was performed on RNA-seq data from two staged testis samples in *Ae. aegypti* and four staged testis samples in *C. capitata*, along with gonadectomised male, ovary and female samples to identify genes with testis-specific splice forms.

Candidate testis-specifically spliced genes were identified from the total set of predicted genes by running a custom Python script on the output of the standard TopHat-Cufflinks-Cuffdiff RNA-seq analysis pipeline, and applying various filtering steps (described below) to maximise sensitivity whilst removing unsuitable genes and minimising false positives. These filtering steps may exclude some valid genes, but for the intended downstream application it is not necessary to identify all testis-specifically spliced genes; it was more important to minimise false positives.

An expression level of 10 FPKM (in the early sample for *Ae. aegypti* and the early spermatocytes sample for *C. capitata*) was chosen as a threshold for the predicted testis-specific splice forms, using the same rationale as discussed for the candidate testis-specifically expressed genes. The threshold for expression of predicted testis-specific splice forms in tissues other than testis was not set at zero, to allow for some noise in the data, but rather at 0.4 FPKM, based on quantification of the known testis-specifically spliced transcripts from the genes *achi* and *vis* in a *D. melanogaster* dataset (data not shown). It was also required that at least one other splice form of the gene was expressed in at least one other sample (gonadectomised male, ovary or female) at a level of 10 FPKM or above, to distinguish testis-specific splicing from testis-specific expression.

In addition to the above expression thresholds, a threshold for exon-exon junction coverage was set to minimise false positives; only introns with more than 10 reads spanning the exon-exon junction were taken forward. False positives may also arise due to low coverage in a particular sample, causing incorrect assembly of a transcript in this sample, for example with a few nucleotides missing at the end, and giving the appearance of alternative splicing. To minimise this source of error, the only introns taken forward were those differing by more than 20 bp at one end at least from introns in other transcripts from the same gene. Finally, only candidates for which the predicted testis-specific intron was within an annotated gene were taken forward, to avoid false positives that are in fact intergenic regions but predicted as introns due to incorrect merging of transcripts during assembly. Using these parameters, 27 and 33 candidate testis-specifically spliced genes were identified for *Ae. aegypti* and *C. capitata* respectively.

**Fig. 6:**
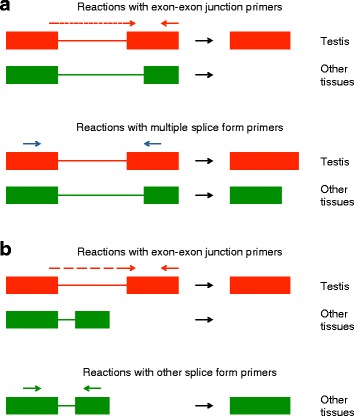
RT-PCR testing of candidate testis-specifically spliced genes. Expression of the predicted testis-specific splice form was assessed using primers designed to span the predicted testis-specific exon-exon junction. Expression of other splice forms was assessed using additional primers targeting either multiple splice forms – both the predicted testis-specific splice form and other splice forms – but yielding products of different sizes (**a**), or other splice forms only (**b**). Note that primers amplifying splice forms other than the predicted testis-specific splice form may still yield a product in testis samples, as these splice forms may be expressed in the testis in addition to the testis-specific splice form. The splice forms illustrated here are simplified examples

Experimental validation of testis-specific splicing required distinguishing between splice forms using RT-PCR. The primer design strategy used is illustrated in Fig. 6. The specificity of the predicted testis-specific splice forms was tested using primers spanning the predicted testis-specific exon-exon junction. Candidates for which primers could also be designed common to both predicted testis-specific and other splice forms were preferred; these allowed additional testing of testis-specificity of the predicted testis-specific splice form, as they should yield products of different sizes in testis and other tissues (Fig. 6a). There were only a small number of these, so all were taken forward for experimental testing. There were further candidates for which primers common to both predicted testis-specific and other splice forms could not be designed (Fig. 6b); for each species the top five of these candidates in order of ascending intron size were taken forward for experimental validation. For *C. capitata*, one additional candidate was also taken forward, as a homologue of the gene was identified as a candidate in *Ae. aegypti*; as mentioned above, candidates that are conserved between species may simplify construct generation in different species. Lists of the candidate genes tested, and the annotated loci that they correspond to, if any, can be seen in Additional file 2.

### Experimental testing of candidate testis-specifically spliced genes

#### RT-PCR

RT-PCR for the selected candidates was performed on testis, gonadectomised male, ovary and gonadectomised female samples, to confirm that the candidates were testis-specifically spliced. The primer design strategy used is illustrated in Fig. 6.

**Fig. 7:**
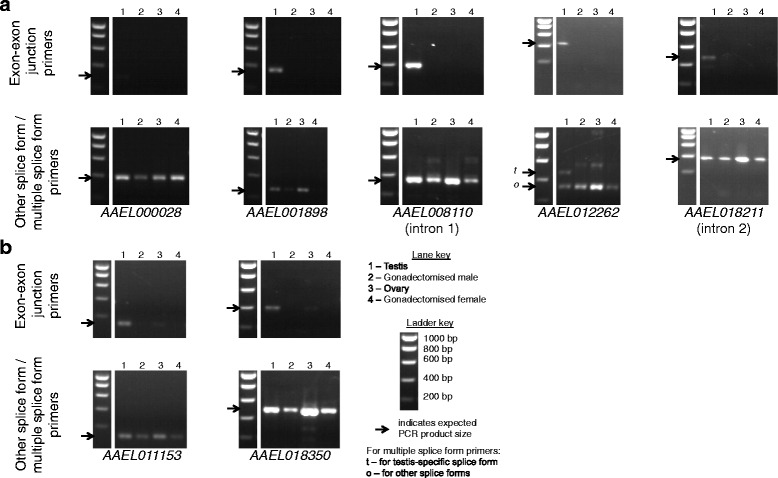
Gels showing PCR results for *Ae. aegypti* splicing candidates. **a** Candidates for which no band of the expected size for the predicted testis-specific splice form could be seen in non-testis samples. **b** Candidates for which a band of the expected size for the predicted testis-specific splice form could be seen in a non-testis sample, but it was only faint. Expected PCR product sizes are indicated with arrows. Bands of unexpected sizes may represent other splice forms that were not predicted, or non-specific amplification

**Fig. 8:**
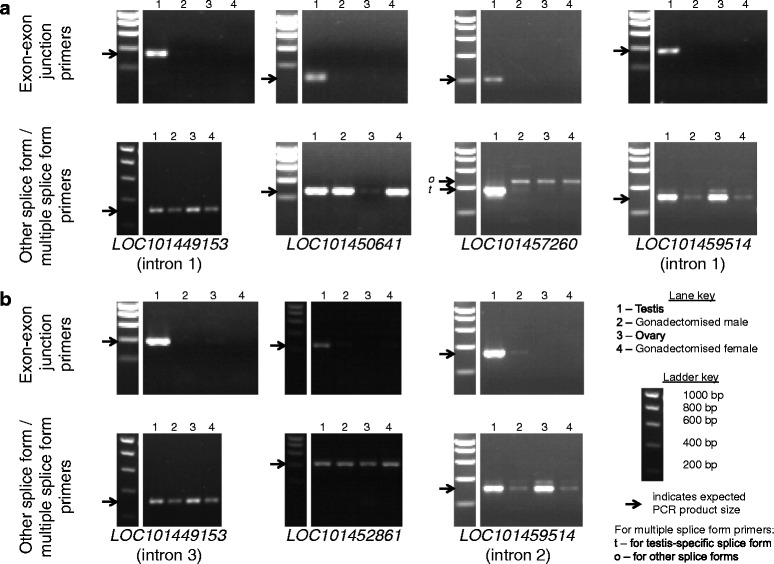
Gels showing PCR results for *C. capitata* splicing candidates. Presented as for Fig. 7

The PCR results varied between candidate genes. For some candidates the predicted testis-specific splice form was not detected, for others it was detected in samples other than testis, and for others no splice forms at all were detected in samples other than testis, suggesting that the gene is testis-specifically expressed rather than differentially spliced. However, the results supported the prediction of testis-specific splicing for some candidates (Figs. 7 and 8), discussed below.

Five *Ae. aegypti* candidate introns (Fig. 7a), within the annotated loci *AAEL000028*, *AAEL001898*, *AAEL008110*, *AAEL012262* and *AAEL018211*, and four *C. capitata* candidate introns (Fig. 8a), within the annotated loci *LOC101449153*, *LOC101450641*, *LOC101457260* and *LOC101459514*, displayed the expected outcome of a positive PCR result for the predicted testis-specific splice form in testis only, and a positive PCR result for other splice forms in other tissues. These nine candidates were taken forward for further testing. Two additional *Ae. aegypti* candidate introns (Fig. 7b), within the annotated loci *AAEL011153* and *AAEL018350*, and three additional *C. capitata* candidate introns (Fig. 8b), within the annotated loci *LOC101449153*, *LOC101452861* and *LOC101459514*, were also taken forward despite positive PCR results for the predicted testis-specific splice form in non-testis samples, as the quantity of product from the non-testis samples was low. Candidates with a low expression in non-testis tissues of the putative testis-specific splice form relative to other splice forms could potentially still be useful for the intended application.

#### qRT-PCR

**Fig. 9:**
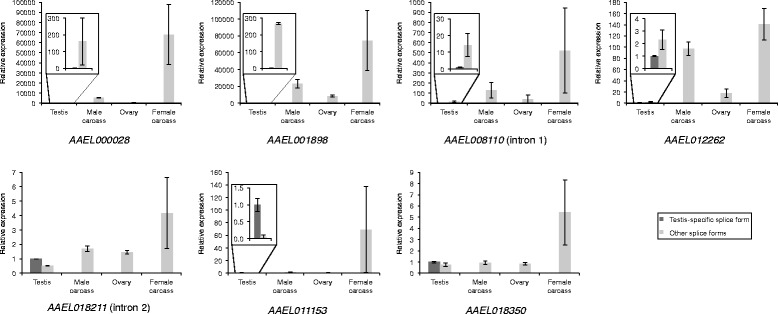
Relative expression levels in different tissues for predicted testis-specific and other splice forms of *Ae. aegypti* splicing candidates, determined using qRT-PCR. Where expression levels in the testis are too low to view at the same scale as for the other splice forms, results for testis are shown inset. The relative expression value of the testis-specific splice form is set at 1 in all cases. Error bars show +/− standard error of the mean for two technical replicates

**Fig. 10:**
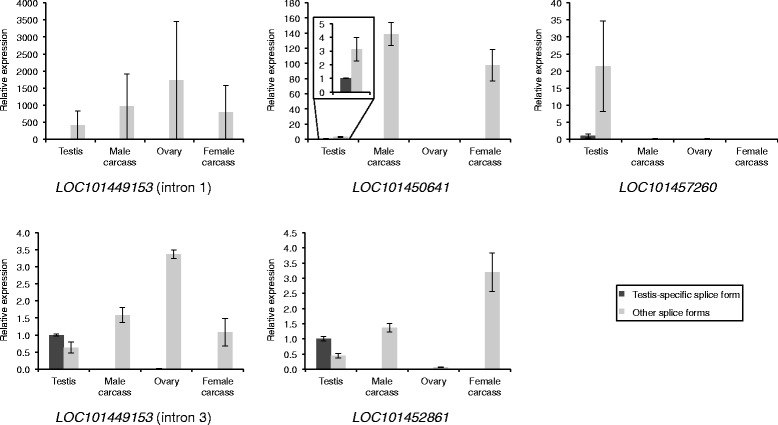
Relative expression levels in different tissues for predicted testis-specific and other splice forms of *C. capitata* splicing candidates, determined using qRT-PCR. Presented as for Fig. 9

The suitability of a testis-specific intron for use in a synthetic construct as discussed above will be affected by the proportions of different splice forms for the corresponding gene in the testis. There may be other splice forms expressed in the testis in addition to the testis-specific splice form. If used to direct testis-specific expression of a coding region, the higher the proportion of the testis-specific splice form compared to other splice forms, the higher the proportion of primary transcripts processed into the splice variant of interest (the testis-specific splice variant). If most transcripts are not of the testis-specific splice form and retain the testis-specific intron, there may be insufficient production of functional transgene product. In order to determine splice form proportions in the testis for the candidates taken forward for further testing, qRT- PCR was performed (Figs. 9 and 10). Gonadectomised male, ovary and gonadectomised female samples were also used in the qRT-PCR to determine the expression level of the predicted testis-specific splice form in these tissues, if any, relative to the expression level of other splice forms. While complete absence of expression of the predicted testis-specific splice form in non-testis tissues would be preferred, candidates with a low level of non-testis expression of the predicted testis-specific splice form relative to other splice forms may still be usable for synthetic biology applications, particularly in combination with other strategies, such as use of testis-specific regulatory regions, for restricting expression to the testis.

The qRT-PCR for the *C. capitata* candidate introns within the annotated locus *LOC101459514* failed to produce meaningful results, with calculations suggesting negative expression of some splice forms, so these introns were excluded. Based on the qRT-PCR results for the other candidates, the estimated proportion of the testis-specific splice form out of all splice forms in the testis ranged from 0.4 to 95% in *Ae. aegypti* (Fig. 9) and 0.24–69% in *C. capitata* (Fig. 10). Candidates at the lower ends of these ranges are unlikely to be suitable for use in a synthetic construct. For example, the results suggest that for *AAEL001898*, only 0.4% of mature transcripts in the testis would retain the intron, and thus only 0.4% of transcripts would be of the desired form if this intron were used to direct testis-specific expression of a coding region. However, candidates at the higher ends of the ranges are more likely to be suitable, and will be taken forward for testing in synthetic constructs. In some cases the qRT-PCR results suggested expression of the testis-specific splice form in non-testis samples, but this was mostly at a very low level (<1%) relative to the expression of other splice forms in these tissues. For the *C. capitata* candidates *LOC10450641* and *LOC101457260* the results suggested that 17–100% of the splice forms in non-testis samples were actually the predicted testis-specific splice form. However, the expression of all splice forms in these non-testis samples was low compared to expression in the testis, so relative errors in quantification are likely to be higher.

### Inter-species conservation

To determine whether any of the candidates we identified were conserved between species, tBLASTx searches were performed, using the candidate sequences from one species as queries and all transcripts predicted by Cufflinks from the other species as a database. A *D. melanogaster* dataset was also used as a database, to provide further confidence in conservation, and also because more supporting information is available on *D. melanogaster* genes.

These BLAST searches revealed one set of homologous testis-specifically expressed candidates and one set of homologous testis-specifically spliced candidates, with conservation between all three species in each case. The *Ae. aegypti* testis-specifically expressed candidate corresponding to the annotated locus *AAEL009267* and the *C. capitata* testis-specifically expressed candidate corresponding to the annotated locus *LOC101459316* are homologous, and both show homology to a *D. melanogaster* gene, *CG7691*, that was also identified as testis-specifically expressed, with higher expression early in spermatogenesis. *AAEL009267* is annotated as a hypothetical protein, while *LOC101459316* and *CG7691* are predicted zinc finger proteins. The expression timing of *AAEL009267* could not be confirmed due to a failed qRT-PCR, but higher expression of *LOC101459316* in early spermatogenesis was confirmed. The *Ae. aegypti* testis-specifically spliced candidate corresponding to the annotated locus *AAEL008110* (*centrosomin*) and the *C. capitata* testis-specifically spliced candidate corresponding to the annotated locus *LOC101449153* (*centrosomin-like*) are homologous, and both show homology to the *D. melanogaster* gene for centrosomin, which is involved in centrosome assembly and is known to have a role in spermatogenesis and display testis-specific splicing in this species [36]. However, it should be noted that qRT-PCR results suggested low abundance in testis of the predicted testis-specific splice form compared to other splice forms for both *AAEL008110* and *LOC101449153*, and thus they may not be suitable for use in synthetic constructs for the reasons discussed above.

## Discussion

In this study, we have used RNA-seq data to identify testis-specifically expressed and spliced genes in the disease vector *Ae. aegypti* and the agricultural pest *C. capitata*. This genome-wide approach represents an advance on previous efforts to find regions for use in insect control constructs, which attempted to identify candidates on an individual basis, often based on distant homology to *D. melanogaster* genes. Using testis samples corresponding to different developmental stages gave sufficient resolution to select testis-specifically expressed genes with expression levels highest in early spermatogenesis, likely to be useful for pre-meiotic protein expression in bipartite synthetic genetic systems such as those involving GAL4-UAS or tTA-tetO.

To identify testis-specific splicing, we have developed a novel computational pipeline for this type of analysis. Whilst the majority of the work is performed by the pre-existing Tuxedo suite of programs, these do not produce finished analyses with regards to alternative splicing, but rather an intermediate output that requires further computation to produce user-friendly candidate lists and sequences. Our pipeline combines this software with custom-written Python scripts to achieve this. Unlike other methods for identifying differential splicing from RNA-seq data [37], it identifies splice forms generated by all types of splice event, not only exon skipping. The outputs are particularly tailored for subsequent experimental testing, containing intron flanking sequences along with numbered exon-exon junction positions to facilitate PCR primer design, and alignments for all transcripts of each gene. In addition to its application here to identify testis-specific splicing, the pipeline could be applied to other sample sets, for example to identify splice forms specific to other tissues, developmental stages, disease states or external conditions.

Based on RNA-seq analysis, we identified a number of candidate testis-specifically expressed genes with expression highest in early spermatogenesis – 57 in *Ae. aegypti* and 68 in *C. capitata*. These comprised a minority of the total number of testis-specifically expressed genes with expression in early spermatogenesis – 388 for *Ae. aegypti* and 667 for *C. capitata*, suggesting that most testis-specifically expressed genes do not exhibit suitable expression timing, and so using samples with sufficient time resolution as we have done is important in identifying suitable candidates for some types of synthetic control systems. We also identified a number of candidate testis-specifically spliced genes – 27 in *Ae. aegypti* and 33 in *C. capitata*. Testing the top candidates with RT-PCR validated the expression profiles of six *Ae. aegypti* and four *C. capitata* testis-specifically expressed genes, and seven *Ae. aegypti* and five *C. capitata* testis-specifically spliced genes, although the testis-specifically spliced genes may not all have suitable splice form ratios. The suitability of these candidates for any particular application should be confirmed with functional testing.

Our findings complement those of Akbari et al. [38], who identified regulatory regions specific to the female germline in *Ae. aegypti*. These regions could be used to direct ovary-specific expression in strategies such as *Medea* and UD^MEL^, which have been shown to be capable of driving population replacement in *Drosophila* [39, 40], while the testis-specific regulatory regions that we have identified could be used in alternative strategies such as sex distortion and paternal effect systems. Testis-specific expression could also be achieved with the testis-specific introns that we have identified. These could be used to achieve testis-specific expression on their own or in combination with testis-specific regulatory elements, or even with regulatory elements active in the testis but not testis-specific. This would allow a wider choice of regulatory elements. The genes that we have identified would also provide a choice of expression levels in a synthetic construct, given the varying expression levels for the testis-specifically expressed genes and varying splice form ratios for the testis-specifically spliced genes. This may be useful as different applications utilising testis-specific expression may require different expression levels.

Some of the genes display testis-specific expression or splicing that is consistent between *Ae. aegypti*, *C. capitata* and *D. melanogaster*. Conserved genes such as this can be particularly useful; they can simplify construct generation across different species, as it is possible that the same or similar sequences may be used for multiple species. Conservation of a regulatory element does not necessarily imply that it will function in the same way across species; there are examples where regulatory elements from one species have failed to drive transgene expression with the same strength or specificity in another species as in the native species, despite the presence of orthologous elements in the non-native species. For example, the *D. melanogaster Actin-5C* promoter was much less active in a transient expression assay in the cricket *G. bimaculatus* than the native actin promoter [41], and displayed a more restricted tissue distribution in transformed *Ae. aegypti* than in *D. melanogaster* [42]. Regulatory elements from the *D. melanogaster* gene *sry-α* failed to drive expression in *C. capitata* [43], and in an example of attempted testis-specific expression, regulatory elements from the *vasa* gene in *An. gambiae* failed to drive expression in *Ae. aegypti* [38]. However, there are many cases of successful inter-species function of regulatory elements for driving targeted transgene expression in insect control systems. For example, the *Ae. aegypti* and *Ae. albopictus Actin-4* promoters have been used interchangeably to generate a female-specific flightless phenotype in both species [12], and the *An. gambiae β2-tubulin* promoter has been used to drive testis-specific expression in an *An. stephensi* transgenic sexing strain [19]. Inter-species functionality of alternative splicing has also been demonstrated; female-specific lethality was achieved in olive fly and *D. melanogaster* using the *C. capitata* sex-specifically spliced *tra* intron [9] and in diamondback moth using the pink bollworm sex-specifically spliced *dsx* intron [10]. Even if inter-species function is not conserved, identifying conserved genes that share a feature of interest facilitates a candidate gene approach to isolating an endogenous element with the desired characteristics.

A potential disadvantage of conserved sequences is that they might also function in non-target species. Transfer to non-target species could theoretically occur by hybridisation or horizontal gene transfer, though for insect species to be able to form fertile hybrids they would need to be very closely related, and most molecular elements from one would likely function to some degree in the other. Horizontal gene transfer between divergent insect species is extremely rare, though detectable over evolutionary timescales for transposons, for example. The consequences of such hypothetical transfer would vary considerably by application, being potentially more significant for highly invasive gene drive systems, much less so for self-limiting strategies such as male-sterile systems. Our approach allows the isolation of both more- and less-conserved sequences from a species of interest, as appropriate.

## Conclusions

In this study we have used RNA-seq data to identify a number of genes with testis-specific expression or splicing potentially suitable to provide molecular components for use in synthetic control systems involving manipulation of the male germline. Some genes displayed conservation of expression or splicing behaviour across species; these may be particularly promising candidates for further investigation. Overall, our findings provide the beginnings of a comprehensive toolkit for male germline expression in synthetic control systems for pest insects.

## Methods

### Insects

*Ae. aegypti* of the Asian wild-type strain (originating from Jinjang, Selangor, Malaysia, colonised by the Institute of Medical Research (Kuala Lumpur, Malaysia) in 1975, from which a colony at Oxitec was established in 2003) [44] were reared under standard conditions, at 27 +/−2°C and 70 +/−10% relative humidity with a 12:12 h light:dark cycle. Larvae were reared in trays and fed with Tetramin® (Tetra GmbH, Germany). Males and females for experiments were separated as pupae. Adults were maintained in cages with *ad libitum* access to a 10% sucrose solution supplemented with 14 U mL^−1^ penicillin and 14 μg mL^−1^ streptomycin (Sigma-Aldrich, UK). Adult females for both colony maintenance and experimental work were fed defibrinated horse blood (TCS Biosciences Ltd., UK) 3–5 days after eclosion.

*C. capitata* of the Toliman wild-type strain (originating from Guatemala, colonised in 1990) were reared under standard conditions, at 26 +/− 1°C and 65 +/− 10% relative humidity with a 12:12 h light:dark cycle. Larvae and adults were kept in plastic containers with *ad libitum* access to a *Drosophila* diet containing maize meal, sucrose and yeast. Pupae were allowed to eclose in a Petri dish containing sand. Males and females were separated shortly after eclosion, before mating.

### RNA extraction and cDNA synthesis

**Fig. 11:**
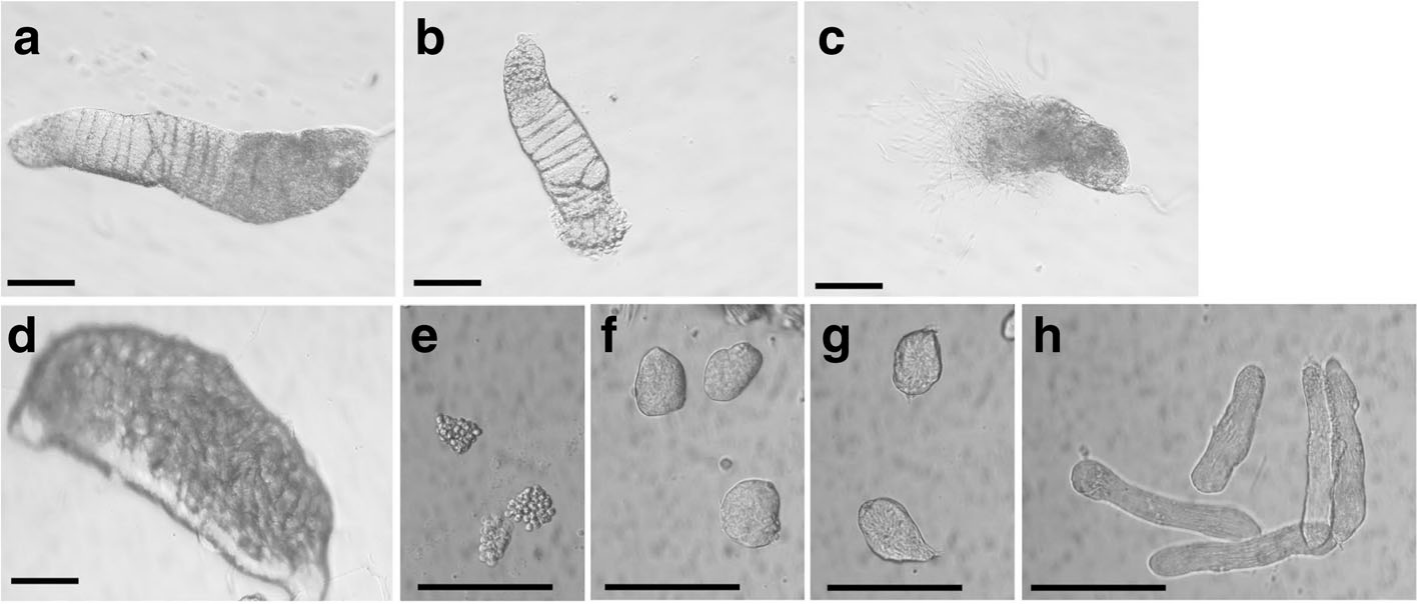
Microscope images illustrating preparation of staged testis samples. **a** Whole testis from *A. aegypti* pupa. **b** Apical region of *A. aegypti* testis after bisection; used to generate “early” sample. **c** Basal region of *A. aegypti* testis after bisection; used to generate “late” sample. **d** Whole testis from *C. capitata* pupa. **e** Isolated *C. capitata* early spermatocytes. **f** Isolated *C. capitata* late spermatocytes. **g** Isolated *C. capitata* early spermatids. **h** Isolated *C. capitata* late spermatids. Scale bar is 100 μm in all panels

For RNA sequencing, total RNA was extracted from staged testis samples, prepared from 3 day old virgin males dissected in phosphate-buffered saline. Tissues from multiple individuals were pooled for each sample. For *Ae. aegypti*, two staged testis samples (referred to as “early” and “late”) were prepared by bisecting testes; the apical region contains cysts of male germline cells in earlier stages of development, up to late spermatocytes and the basal region contains spermatid cysts in later stages of development (Fig. 11a–c). Both of these samples also contained somatic cells from the testis sheath. An *Ae. aegypti* gonadectomised male sample was also prepared from the same males used for the testis samples. For *C. capitata*, four staged testis samples were prepared – early spermatocytes, late spermatocytes, round spermatids and elongated spermatids – by spilling cysts out of the testes and examining isolated cysts with a Nikon Eclipse Ti-S inverted microscope (Fig. 11d–h). Cysts at specific stages were identified based on cell size and morphology, and collected manually with a pulled-out Pasteur pipette. A *C. capitata* ovary sample was also prepared, from 5 day old virgin females dissected in phosphate-buffered saline. Total RNA was extracted using TRIzol® (Life Technologies Ltd, Paisley, UK), according to the manufacturer’s instructions. The samples used for RNA-seq are summarised in Table 1. Microscope images illustrating testis dissections are shown in Fig. 11.

**Table 1:**
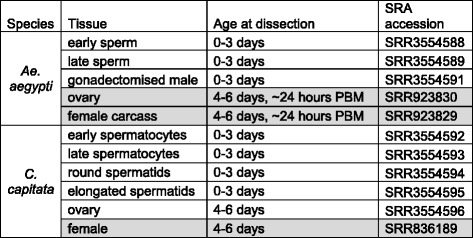
Samples used for RNA-seq analysis

Samples from other studies for which data were downloaded from the SRA are highlighted in grey

*PBM* post-blood meal

For RT-PCR, total RNA was extracted from testis and gonadectomised male samples, prepared from 0 to 3 day old virgin males, and from ovary and gonadectomised female samples, prepared from 4 to 6 day old virgin females. *Ae. aegypti* females were dissected approximately 24 h post-blood meal (PBM). For qRT-PCR, total RNA was also extracted from staged testis samples, prepared as described above for RNA sequencing, except in this instance only two samples – spermatocytes and spermatids – were prepared for *C. capitata*. Tissues from multiple individuals were pooled for each sample. Samples were either stored in RNALater (Qiagen, Manchester, UK) or lysis buffer (Life Technologies Ltd, Paisley, UK or Norgen Bioteck Corp., Ontario, Canada) at −20°C until RNA extraction, or RNA was extracted immediately. Total RNA was extracted using either a Norgen Total RNA Purification Kit (Norgen Biotek Corp., Ontario, Canada) or an Ambion RNAqueous Kit (Life Technologies Ltd, Paisley, UK) according to the manufacturer’s instructions. cDNA for PCR was synthesised using a RevertAid First Strand cDNA Synthesis Kit (Thermo Scientific, Pittsburgh, USA) with random hexamer primers according to the manufacturer’s instructions. The samples used for RT-PCR are summarised in Table 2.

**Table 2:**
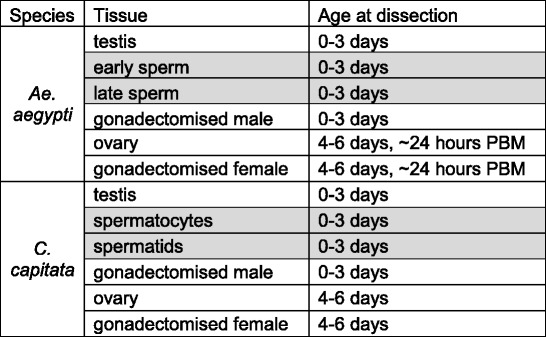
Samples used for RT-PCR analysis

Additional samples used for qRT-PCR analysis only are highlighted in grey

*PBM* post-blood meal

### RNA sequencing

Library preparation, including polyA selection, was performed using an Illumina TruSeq RNA Sample Preparation Kit (Illumina, UK), according to the manufacturer’s instructions. Sequencing was performed by the NGS facility at Glasgow Polyomics (University of Glasgow, UK) using the Illumina Genome Analyzer II platform, with single reads of 73 nucleotides.

### Data from other studies

RNA-seq data from other studies were downloaded from the SRA [34]. This comprised data from a *C. capitata* female sample, and data from *Ae. aegypti* ovary and gonadectomised samples, published in Akbari et al. (2013) [31]. The samples used are summarised in Table 1.

### Sequence data processing

The overall quality of the sequencing reads was assessed using FastQC (v0.10.1) [45]. Raw reads were processed to remove adapter sequence using FASTA/Q Clipper from the FASTX_Toolkit [46] and sequences of poor quality using Sickle [47]. Reference indexes of the *Ae. aegypti* genome assembly AaegL2 [48] (obtained from VectorBase) and the *C. capitata* genome assembly Ccap_1.0 [49] (obtained from NCBI) were constructed using Bowtie2 [50]. Trimmed reads were aligned to these indexes using TopHat2 (v2.0.9) [51]. Transcript assemblies were created from the alignments using the reference annotation based transcript assembly method [52] with Cufflinks (v2.1.1) [53] followed by Cuffmerge (v1.0.0) [54]. Transcript expression in each sample was quantified using Cuffdiff2 (v2.1.1) [55].

### Identification of candidate testis-specifically expressed and spliced genes

**Fig. 12:**
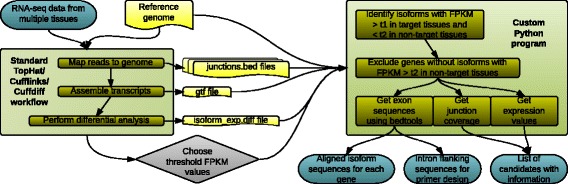
Computational pipeline for identification of candidate testis-specifically spliced genes. RNA-seq reads were mapped to the relevant reference genome using TopHat. Transcript assemblies were generated using Cufflinks. Transcript expression was quantified using Cuffdiff. The output of these steps, along with user-defined threshold FPKM values, was used as input for a custom Python program. Custom Python scripts in combination with bedtools were used to output a list of candidates with associated information used for further filtering, such as exon-exon junction coverage and expression values, as well as sequences in a convenient format for primer design – intron flanking sequences, and alignments of all splice forms for each gene

Candidate testis-specifically expressed and spliced genes were identified from the output of Cuffdiff2, and their sequences obtained, using custom Python scripts in combination with bedtools (version 2.16.2) [56]. An outline of the use of this pipeline to identify candidate testis-specifically spliced genes is illustrated in Fig. 12, and the documentation for the Python scripts is provided in Additional file 3. Filtering steps were applied as described in the results section. For steps requiring alignment of sequences, Geneious (7.0.5) [57] was used.

For the candidate testis-specifically expressed genes, only genes with higher expression in the samples from early stages of spermatogenesis (the early sample for *Ae. aegypti*; the early spermatocytes sample for *C. capitata*) than in the samples from the later stages (the late sample for *Ae. aegypti*; the mean expression in the late spermatocytes, round spermatids and elongated spermatids samples for *C. capitata*) were taken forward.

### Inter-species comparison

To determine whether candidates were conserved between species, BLAST analysis was performed. Sequences of all transcripts predicted by Cufflinks were extracted using a custom Python script in combination with bedtools (version 2.16.2) [56]. BLAST databases were created from these sequences using the makeblastdb tool. tBLASTx searches were then performed using the transcript sequences from one species to query a BLAST database from another species. A threshold E value of 0.001 was used.

### Experimental testing of candidates

RT-PCR primers were designed using Primer-BLAST [58]. RT-PCR was performed on a TGradient thermocycler (Biometra, Goettingen, Germany) using a PCRBIO kit (PCR Biosystems Ltd, London, UK), according to the manufacturer’s instructions. The reaction parameters were: 95°C for 30 s, 2 cycles of 95°C for 15 s, 55°C for 30 s and 72°C for 2 min, 33 cycles of 95°C for 15 s, 55°C for 15 s and 72°C for 30 s, and finally 72°C for 1 min. Reactions with primers targeting *RpL22* transcripts were performed as positive controls. RT-PCR products were visualized on 1.5–2% agarose gels.

qRT-PCR was performed on an Mx3500P instrument (Stratagene, La Jolla, USA) using iQ™ SYBR® Green Supermix (Bio-Rad, Hemel Hempstead, UK) according to the manufacturer’s instructions. Reactions were performed with serial dilutions to determine primer efficiency. Reactions with primers targeting *α-tubulin* and 18S rRNA transcripts in *Ae. aegypti* and *α-tubulin* and *Rps17-like* transcripts in *C. capitata* were performed for normalisation. The reaction parameters were: 95°C for 5 min, followed by 40 cycles of 95°C for 15 s, 55°C for 15 s and 60°C for 15 s.

For the candidate testis-specifically expressed genes, expression was calculated relative to an expression level in the early (for *Ae. aegypti*) or early spermatocytes (for *C. capitata*) samples of 1000 for the geometric mean of the two genes used for normalisation. For the candidate testis-specifically spliced genes, expression was calculated relative to an expression level in the testis of 1 for the predicted testis-specific splice form.

To confirm that the PCR results reflected the predicted candidates, PCR products were purified using a QIAquick PCR Purification Kit (Qiagen, Manchester, UK) and sent for sequencing by GATC Biotech (Cologne, Germany).

PCR primer sequences are available in Additional file 4.

## Additional files

Additional file 1: Sequencing and alignment statistics. Table of sequencing and alignment statistics. (XLSX 47 kb)
Additional file 2: Candidate genes tested experimentally. Tables of candidate *Ae. aegypti* and *C. capitata* testis-specifically expressed and testis-specifically spliced genes tested experimentally. (XLSX 42 kb)
Additional file 3: Documentation for Python scripts. Documentation for custom Python scripts used to identify candidate testis-specifically expressed and spliced genes. (PDF 178 kb)
Additional file 4: Primer sequences. List of primer sequences used in the study. (PDF 90 kb)

## Abbreviations

*Ae.*: *Aedes*
*An.*: *Anopheles*
*C.*: *Ceratitis*
*D.*: *Drosophila*
FPKM: Fragments per kilobase per million reads
Kb: Kilobase
PBM: Post-blood meal
PCR: Polymerase chain reaction
qRT-PCR: Quantitative reverse transcriptase polymerase chain reaction
RT-PCR: Reverse transcriptase polymerase chain reaction
tTA: Tetracycline-repressible transactivator
UAS: Upstream activating sequence
