# Supplementary material for: Identification of genes for engineering the male germline of Aedes aegypti and Ceratitis capitata
Source: BMC Genomics. 2016 Nov 21;17:948. doi: 10.1186/s12864-016-3280-3 (PMC5117610; doi:10.1186/s12864-016-3280-3)
Supplement: Additional file 3: — Documentation for Python scripts. Documentation for custom Python scripts used to identify candidate testis-specifically expressed and spliced genes. (PDF 178 kb) [file 12864_2016_3280_MOESM3_ESM.pdf]

RNA-seq_analysis_tools

About

RNA-seq_analysis tools is a package of Python scripts designed primarily to analyse differential expression and splicing between samples using RNA-seq data. There are also scripts for obtaining gene/transcript sequences and co-ordinates. The scripts work on the output of the Tuxedo suite of tools for RNA-seq analysis (Trapnell *et al.*, 2012) to identify genes, transcripts, or introns in splice forms that are expressed above a user-specified threshold in one or more user-specified samples and at or below a user-specified threshold in one or more other user-specified samples.

Citation

To cite RNA-seq_analysis_tools or any of the individual scripts please cite this paper.

Requirements

All the scripts in RNA-seq_analysis_tools require Python. They have been tested only with Python 2.7.3. Some of the scripts have further requirements; see the documentation on the individual scripts below for more information.

Differential_expression

About

Differential_expression is a combination of two Python scripts for analysing differential expression between samples using RNA-seq data. These scripts work on the output of the Tuxedo suite of tools for RNA-seq analysis(Trapnell *et al.*, 2012) to identify genes or transcripts that are expressed above a user-specified threshold in one or more user-specified samples and at or below a user-specified threshold in one or more other user-specified samples. Genes are identified with `differential_expression_genes.py` and transcripts are identified with `differential_expression_transcripts.py`.

For example, Differential_expression could be used to identify putative male-specifically expressed genes, which are expressed in males, but not in females. It may be advisable to use a threshold higher than 0 FPKM to detect genuine expression of genes, to reduce false positives, and to use a threshold higher than 0 FPKM to confirm genuine absence of expression of genes, to reduce false negatives. False positives or false negatives could be caused by e.g. errors in transcript assembly or sample cross-contamination. So in this case the user might specify a threshold of 10 FPKM for male samples (genes must have an expression level above 10 FPKM in these samples), and 0.5 FPKM for female samples (genes must not an expression level above 0.5 FPKM in these samples).

The information output by Differential_expression is already available in the output of Cuffdiff, but not in a convenient format; Differential_expression extracts only the genes of interest and lists information on these genes in a convenient tabular format.

Requirements

Differential_expression requires Python. It has been tested only with Python 2.7.3.

Differential_expression works on the output of the Tuxedo suite of tools for RNA-seq analysis. A file output by Cuffdiff is required. It has been tested only with Cuffdiff v2.1.1.

Usage

Both Differential_expression scripts have a number of required and optional arguments, detailed below.

Required arguments

- `above_threshold` - space-separated list of samples for which genes/transcripts output must have an FPKM value above `t` (see 'Optional arguments' section); the sample names must be written exactly as in the isoform_exp.diff file (see below)
- `absent` - space-separated list of samples for which genes/transcripts output must not have an FPKM value above `t_absent` (see 'Optional arguments' section); the sample names must be written exactly as in the isoform_exp.diff file (see below)
- `expression` - path to isoform_exp.diff file output by Cuffdiff

Optional arguments

- `t` - threshold FPKM value - genes/transcripts output must have an FPKM value above this in samples listed in the `above_threshold` argument; default value is 0
- `t_absent` - threshold absent FPKM value; genes/transcripts output must not have an FPKM value above this in samples listed in the `absent` argument; default value is 0
- `other` - space-separated list of samples for which expression information is desired but for which expression values do not influence the genes/transcript output; the sample names must be written exactly as in the isoform_exp.diff file

Example

To identify putative male-specifically expressed genes as per the example in the 'About' section, the command might be:

```
./differential_expression_genes.py -t 10 -t_absent 0.5 -above_threshold
male1 male2 -absent female1 female2 -expression isoform_exp.diff
```

Output

Both Differential_expression scripts produce one output file, called `gene_expression_profiles.txt` for`differential_expression_genes.py`, and `transcript_expression_profiles.txt` for `differential_expression_transcripts.py`. This file lists the genes/transcripts identified as fitting the user-specified criteria (e.g. male-specifically expressed) and their expression values in the user-specified samples in tabular format.

Differential_splicing

About

Differential_splicing is a Python script for analysing differential splicing between samples using RNA-seq data. It works on the output of the Tuxedo suite of tools for RNA-seq analysis (Trapnell *et al.*, 2012) to identify introns in splice forms that are expressed above a user-specified threshold in one or more user-specified samples and at or below a user-specified threshold in one or more other user-specified samples.

For example, Differential_splicing could be used to identify putative male-specific introns, which are present in splice forms expressed in males, but absent from splice forms of the same genes expressed in females. It may be advisable to use a threshold higher than 0 FPKM to detect genuine presence of splice forms, to reduce false positives, and to use a threshold higher than 0 FPKM to confirm genuine absence of splice forms, to reduce false negatives. False positives or false negatives could be caused by e.g. errors in transcript assembly or sample cross-contamination. So in this case the user might specify a threshold of 10 FPKM for male samples (introns must be in transcripts with an expression level above 10 FPKM in these samples), and 0.5 FPKM for female samples (introns must not be in transcripts with an expression level above 0.5 FPKM in these samples).

Requirements

Differential_splicing requires Python. It has been tested only with Python 2.7.3. It also requires bedtools. It has been tested only with bedtools version 2.16.2.

Differential_splicing takes a few hours to run on a standard-sized dataset and so is probably best run on a high performance server with multiple cores.

Differential_splicing works on the output of the Tuxedo suite of tools for RNA-seq analysis. Files output by Cuffdiff, Cuffmerge and TopHat are required. It has been tested only with Cuffdiff v2.1.1, Cuffmerge v1.0.0 and TopHat v2.0.9.

Usage

Differential_splicing has a number of required and optional arguments, detailed below.

Required arguments

- `above_threshold` - space-separated list of samples for which introns output must be in splice forms with an FPKM value above `t` (see 'Optional arguments' section); the sample names must be written exactly as in the isoform_exp.diff file (see below)
- `absent` - space-separated list of samples for which introns output must not be in splice forms with an FPKM value above `t_absent` (see 'Optional arguments' section); the sample names must be written exactly as in the isoform_exp.diff file (see below)
- `genome` - path to genome FASTA file
- `expression` - path to isoform_exp.diff file output by Cuffdiff
- `gtf` - path to merged.gtf file output by Cuffmerge
- `junctions` - space-separated list of paths to junctions.bed files output by Tophat for `above_threshold` and`other` samples, with the paths for the `above_threshold` samples listed first and in the same order as for the`above_threshold` argument, and the paths for the `other` samples listed second and in the same order as for the`other` argument

Optional arguments

- `t` - threshold FPKM value - introns output must be in splice forms with an FPKM value above this in samples listed in the `above_threshold` argument; default value is 0
- `t_absent` - threshold absent FPKM value; introns output must not be in transcripts with an FPKM value above this in samples listed in the `absent` argument, and there must be an alternative transcript for the gene in question with an FPKM value above this in at least one of the samples listed in the `absent` argument (otherwise the program would identify introns in genes with sample-specific expression as well as genes with sample-specific splicing); default value is 0
- `other` - space-separated list of samples for which expression information is desired but for which expression values do not influence the introns output; the sample names must be written exactly as in the isoform_exp.diff file
- `min_specific_intron` - introns output must have a length in nucleotides above this value; default value is 10

Example

To identify putative male-specific introns as per the example in the 'About' section, the command might be:

```
./differential_splicing.py -t 10 -t_absent 0.5 -above_threshold male1 male2
-absent female1 female2 -genome genome.fa -expression isoform_exp.diff -gtf
merged.gtf -junctions TopHat_files/Male1/junctions.bed
TopHat_files/Male2/junctions.bed TopHatfiles/Female1/junctions.bed
TopHatfiles/Female2/junctions.bed
```

Output

Differential_splicing produces a number of output files:

- `specific_intron_information` - This file lists the splice forms with introns identified as fitting the user-specified criteria (e.g. male-specific) and provides further details in tabular format, as described below. The final three columns provide information useful for primer design for experimental validation. *Note: Introns that are present in multiple different splice forms will be listed multiple times - one line corresponds to one splice form.
  - The first column gives the locus of the gene in the genome.
  - The second, third and fourth columns give information on the gene and transcript ID.
  - The next columns give the expression levels of the splice form in each of the user-specified samples.
  - The next columns give the number of reads spanning the predicted exon-exon junction for the intron in each of the user-specified samples.
  - The next column gives the location in the sequence provided in the final column of the predicted exon-exon junction for the intron in question. This number is the nucleotide on the 3' side of the exon-exon junction. This is useful when designing primers to span this junction, to experimentally validate the predicted intron - for example this value minus 1 can be used for the value of the 'Overlap junctions' parameter of Primer-BLAST to design primers spanning this junction.
  - The next column gives the location in the sequence provided in the final column of any other predicted exon-exon junctions. These may be useful to know as it may be advisable when designing primers to experimentally validate the predicted intron in question to avoid primers spanning other predicted exon-exon junctions, as this may complicate matters and lead to false negative results.
  - The final column gives the transcript sequence surrounding the intron. This is useful for primer design. A maximum of 500 bp either side of the intron is shown, as a typical PCR product is not longer than 1000 bp.
- `intron_co-ordinates.bed` - This file lists the genomic co-ordinates of the introns identified as fitting the user-specified criteria (e.g. male-specific). This is useful for extracting the sequences of the introns from the genome FASTA file (using bedtools).
- `gene_expression_profiles.txt` - This file lists the FPKM values for all transcripts from genes identified as having at least one transcript with an intron fitting the user-specified criteria.
- `Sequences` - This folder contains FASTA files for the genes with introns identified as fitting the user-specified criteria (e.g. male-specific). The file called `all_genes_genomic_sequences` is a multi-FASTA file containing genomic sequences for all the relevant genes. This is useful if you want to BLAST the

genes, e.g. if they are not annotated and you want to know what function they may have. The remaining files in this folder are multi-FASTA files for each individual gene. The first entry in each file is the genomic sequence for the gene. This is followed by the sequence(s) for the transcript(s) identified as having introns fitting the user-defined criteria. This is followed by the sequence(s) for the other transcript(s) for that gene. The transcript sequences have dashes for nucleotides in introns, so that all sequences line up with each other when imported into programs for visualisation (e.g. Geneious).

Get_gene_co-ordinates

About

Get_gene_co-ordinates is a Python script designed for obtaining gene co-ordinates from the output of the Tuxedo suite of tools for RNA-seq analysis (Trapnell *et al.*, 2012) for genes of interest, though it could also be used on gtf files from other sources. This might be useful for e.g. comparing the co-ordinates of predicted genes to those of annotated genes, to identify genes where the annotation may be incomplete.

Requirements

Get_gene_co-ordinates requires Python. It has been tested only with Python 2.7.3.

Usage

Get_gene_co-ordinates has two required arguments, detailed below.

Required arguments

- `gtf` - path to gtf file
- `genes` - path to text file containing list of genes (in a single column) for which transcript sequences are desired

Example

To obtain gene co-ordinates for selected genes, the command might be:

```
./get_gene_co-ordinates.py -gtf merged.gtf -genes genes.txt
```

Output

Get_gene_co-ordinates produces one output file, called `gene_co-ordinates.bed`. This is a BED file containing co-ordinates for all the genes listed in the input text file.

Get_transcript_sequences

About

Get_transcript_sequences is a pair of Python scripts for obtaining transcript sequences from the output of the Tuxedo suite of tools for RNA-seq analysis (Trapnell *et al.*, 2012), though it could also ge used on gtf files from other sources. The `get_all_transcript_sequences.py` script obtains all predicted transcript sequences, which might be useful for e.g. creating a BLAST database to BLAST other sequences against. The`get_transcript_sequences_aligned.py` script obtains predicted transcript sequences in an aligned format for genes of interest, which might be useful for e.g. primer design to test genes of interest.

Requirements

Get_transcript_sequences requires Python. It has been tested only with Python 2.7.3. It also requires bedtools. It has been tested only with bedtools version 2.16.2.

Usage

Both Get_transcript_sequences scripts have two required arguments, and `get_transcript_sequences_aligned.py` has one additional argument, detailed below.

Required arguments

- `genome` - path to genome FASTA file
- `gtf` - path to gtf file

Additional argument for `get_transcript_sequences_aligned.py`:

- `genes` - path to text file containing list of genes (in a single column) for which transcript sequences are desired

Example

To obtain sequences for all transcripts predicted in the gtf file, the command might be:

```
./get_all_transcript_sequences.py -gtf merged.gtf -genome genome.fa
```

To obtain aligned transcript sequence for selected genes, the command might be:

```
./get_transcript_sequences_aligned.py -gtf merged.gtf -genome genome.fa -
genes genes.txt
```

Output

The `get_all_transcript_sequences.py` script produces one output file, called `all_transcripts.fa`. This is a multi-FASTA file containing sequences for all the transcripts predicted in the gtf file.
The `get_transcript_sequences_aligned.py` script produces multiple output files, one for each gene listed in the input text file. Each is a multi-FASTA files containing sequences for the genomic sequence followed by all the transcripts predicted in the gtf file for that gene. The transcript sequences have dashes for nucleotides in introns, so that all sequences line up with each other when imported into programs for visualisation (e.g. Geneious).
