# Supplementary material for: Identification of genes for engineering the male germline of Aedes aegypti and Ceratitis capitata
Source: BMC Genomics. 2016 Nov 21;17:948. doi: 10.1186/s12864-016-3280-3 (PMC5117610; doi:10.1186/s12864-016-3280-3)
Supplement: Additional file 4: — Primer sequences. List of primer sequences used in the study. (PDF 90 kb) [file 12864_2016_3280_MOESM4_ESM.pdf]

Primers for expression candidates

RT-PCR primers

Ae. aegypti

Novel gene 1 forward	ACCGAAGCTACAAGGACGC
Novel gene 1 reverse	CAGCAGCCGACCCGGAA
AAEL000463 forward	GGCCGTTTTGGCACATTCTT
AAEL000463 reverse	CACGCGTTTTTCACTTTGCG
AAEL001333 forward	CGAGACCGTTCTCCTCAACC
AAEL001333 reverse	TCCTCGCACATTTTTCTCCG
AAEL003021 forward	GGCCGTTTTGGCACATTCTT
AAEL003021 reverse	CACGCGTTTTTCACTTTGCG
AAEL004293 forward	GCGAGAAAAAGAAGCGTAAAAC
AAEL004293 reverse	TCCTCAGTTGACTCACGTCG
AAEL006665 forward	TCAAAGCCCTCAAGAATCGGA
AAEL006665 reverse	TCATTGCTTGTGCACTCACC
AAEL009267 forward	GTGAAGACGACCTTGCTCCA
AAEL009267 reverse	CCATGTGACGTTCCTTTGCG
AAEL010268 forward	CCTGCACCGATCGAAGTCAA
AAEL010268 reverse	TGGTGGTGGCAGATGAAACA
AAEL011838 forward	GGCAATCGGTTGTACCTGGA
AAEL011838 reverse	ATCACATCGCCAGGCTTGAA
AAEL012065 forward	CGCGAATACCATAAGCCCCT
AAEL012065 reverse	CGCAGCTACCACTACTACCG
AAEL012239 forward	GGTGCTAGTGGTGGAACGAA
AAEL012239 reverse	TCTTTGGCTTCACGCTTTGC
RpL22 forward	AAACAACTCCTGCGAGGCAA
RpL22 reverse	CACGCAGGCTGTTCTTCTTC

C. capitata

LOC101449084 forward	TACTGAGACAACAGCGCCAG
LOC101449084 reverse	TCGAACGCTCCGATACCAAG
LOC101449780 forward	TTGCCGAAACTTGCCCAAAG
LOC101449780 reverse	CACTCAACCGGATCACCCAA
LOC101451111 forward	CAATTTATGGGCCTCTTTCGG
LOC101451111 reverse	CGATTCGCCAACGTAAGCAG
LOC101451785 forward	AGCGTTGGGTTGTGTGATGA
LOC101451785 reverse	GACAATCCCGATGGAGCAGT
LOC101454909 forward	TGCTCATGTTGCTGTTGTGC
LOC101454909 reverse	GCCGATGATGTAGATGCCGA
LOC101455161 forward	GCGGAAGTACTCGGCTATTG
LOC101455161 reverse	CCCGTTGGTAGGACATGCT
LOC101457895 forward	CTACAGCGAGCTACGTCCAT
LOC101457895 reverse	CCGGACCCTCTGCTTTCATT
LOC101459316 forward	GAAAATCGCAAAGCTGCCCA
LOC101459316 reverse	TGATGCTTGCTGGCATCTCA
LOC101459689 forward	CCCACACTGATCGGCTACTC
LOC101459689 reverse	ATGTAGCCTGCAGCAAAGGT
LOC101462854 forward	GTAGTCGTACTCACCGAGCC
LOC101462854 reverse	CGCGCAGTGATTATCTCGTT
RpL22 forward	CAACACGGATCCAATCACGC
RpL22 reverse	TGCTGCTGCATCTGCTACTT

qRT-PCR primers

Ae. aegypti

AAEL001333 forward	CGTACTCCACGGAGGTCATC
AAEL001333 reverse	GTCCTCGCACATTTTTCTCCG
AAEL003021 forward	GGAGCTGCATGAGAGGTGTT
AAEL003021 reverse	CATGCTGACCAGGACCATGA
AAEL006665 forward	TCAAAGCCCTCAAGAATCGGA
AAEL006665 reverse	TCGTTTGATGGCGTACAGGG
AAEL009267 forward	TGAGATTAAGTTCCACGACGAC
AAEL009267 reverse	CCGTTTCAGTGCGACTTGTG
AAEL010268 forward	GCTTTGACAGTGAAACTGTGGC
AAEL010268 reverse	CGCAAATCAGTGAAGCTTGGA
AAEL012065 forward	CGCGAATACCATAAGCCCCT
AAEL012065 reverse	TGGTGGTACGTAACCCGTTG
AAEL012239 forward	GCCAACGGAGATCTAGAAGTT
AAEL012239 reverse	CGCTTTGCCTTGACATGAGC
α-tubulin forward	TCGGACAAAACCATCGGAGG
α-tubulin reverse	ACGGTTGGTTCCAGATCGAC

C. capitata

LOC101449084 forward	TGTTGAGAGCATCACGATCAC
LOC101449084 reverse	TGCCTCATCGTCGGAATGTT
LOC101449780 forward	TTGAAGCTCCCCGATGTTGC
LOC101449780 reverse	TGACCAATAGCACCACTGGC
LOC101451111 forward	CCCAAAGCAGCAGGTAGACA
LOC101451111 reverse	GCATTCACGTCCAATGTCGC
LOC101451785 forward	GACCGTTATGTGCGTCATGC
LOC101451785 reverse	GCCAACGGTGTTCTTCAACG
LOC101457895 forward	TCTGAAACGTACCCAGAAGGC
LOC101457895 reverse	CCCGGTCATATCTGTCAGCC
LOC101459316 forward	CGCTCCATAAGCGTCGTATCT
LOC101459316 reverse	CCCAAACCGATGGTTGAGAA
LOC101459689 forward	AAACGCAAAGCCACCTTGAC
LOC101459689 reverse	TGGGTTGGTGCCACGAATTA
LOC101462854 forward	CCATTGTTAAACGAGGCAACGA
LOC101462854 reverse	TGGCACCATTATACCCTGCG
α-tubulin forward	TGCCCTACCCACGTATTCAC
α-tubulin reverse	ACCATCTGGTTGGCTGGTTC

Primers for splicing candidates

RT-PCR primers

Primers for amplifying both predicted testis-specific and other splice forms

Ae. aegypti

AAEL001916 forward	GGCCGGGCTCTTAATGTGAT
AAEL001916 reverse	GCAACGACAATGGCTGGCAA
AAEL005651 forward	CGTAGTTCGCTTCGTGGAGT
AAEL005651 reverse	ACTAAGCACCGGTTTCTCCG
AAEL005935 forward	CGGGGTCCTTTTACACCGAA
AAEL005935 reverse	GAGAATCAAGTGGTCGAGCG
AAEL007258 forward	CCCGTCGCCTATTTCACCAA
AAEL007258 reverse	GTTTGGGGATAACGTATCGC
AAEL008110 intron 2 forward	CAAATGCATGATGCGTTCCT
AAEL008110 intron 2 reverse	GCTGCAAGCGTTGGATATGG
AAEL008428 forward	GGGACTTTGACTCGCACAGA
AAEL008428 reverse	AGCAGCAATTCGGTTTTGCAT
AAEL010509 forward	TAAATCGCCGCCATCACCAT
AAEL010509 reverse	GATTTAGAGCGGCCGAGTGA
AAEL011105 forward	TCCGAGTGGTCGCCATTTTG
AAEL011105 reverse	TCACGTTGTCCTCATGGGTG
AAEL011968 forward	TCCAACTGTTCAGCTCGTCG
AAEL011968 reverse	TGGCACAGTAAGCCTGTTGA
AAEL012262 forward	CAGTGCCAGCAAAGCAGAAG
AAEL012262 reverse	TACAACCGAGCGTCATTGCT
AAEL013571 forward	GTCGAGAAGGTACTCGAGCG
AAEL013571 reverse	GACGTCGAAGCAACTGGGTT
AAEL014957 forward	TGCACGAAAAAGGAGCAACG
AAEL014957 reverse	AGTTTGTGTAGCGGTGCGTT
AAEL017395 forward	TGATTCGTCGGTTGGCTGTT
AAEL017395 reverse	CACCACTCGTCTGTGTCTCG
AAEL018039 forward	TCAGCAGCAACCGTTCTACC
AAEL018039 reverse	CCGCGTTGAGTCAGGAACAT
AAEL018211 intron 1 forward	CCATCTTTCGACGCGTGTTC
AAEL018211 intron 1 reverse	TCCAAGTCCGCAAGACAAGG
AAEL018350 forward	ATGGTGGCCCAAAACCAATG
AAEL018350 reverse	TTGTTTGTGATGCGGGACTG

C. capitata

LOC101449503 forward	CCGCCCTTTAGGTGTATGGG
LOC101449503 reverse	TGTTTTCGTATTGATTTTCCCTGG
LOC101450565 forward	AACGGGGTTTGGCAACTGTA
LOC101450565 reverse	GAAGGATCGCGTTGAGACCA
LOC101453609 forward	TGAGGAGGGACACAGGTCAA
LOC101453609 reverse	TGCAGCTTCCGATGTCTGTT
LOC101457260 forward	CTCACCACCGGGTGATATGG
LOC101457260 reverse	ACCGCGAGATCCGAAAATGT
LOC101459514 intron 1 forward	GGAGAAGCAACTGGGGTGTT
LOC101459514 intron 1 reverse	TAGGGCAAACTACTGCACCG
LOC101459514 intron 2 forward	TGGCGATCCTGCTCACCGAA
LOC101459514 intron 2 reverse	TAGGGCAAACTACTGCACCG

Primers for amplifying predicted testis-specific splice form

Ae. aegypti

AAEL000028 forward	TTTGGTTGTCCAGGTCCCAC
AAEL000028 reverse	CGTTTTAGTTGGAAAATTGTTTGCT
AAEL001898 forward	TGGGCTAAAAAGAGAGGCAA
AAEL001898 reverse	CCCAGTACATTTAAAAAGCATGAAG
AAEL002083 forward	CAGATTCGTCCTGATCGCCA
AAEL002083 reverse	ATGCTAACAAGCGCTGTCCA
AAEL008110 intron 1 forward	GGCAATCAAACCCTTGATTCTGT
AAEL008110 intron 1 reverse	AATGCAAGTTTCGTCCGTGC
AAEL008110 intron 2 forward	CTTGCTCATTTGCTCAGCATC
AAEL008110 intron 2 reverse	GCTGCAAGCGTTGGATATGG
AAEL008428 forward	GGGACTTTGACTCGCACAGA
AAEL008428 reverse	CGGTTTTGCATGTCTTCATAAGC
AAEL011153 forward	TCTGCCTTGCCAGTGATTGA
AAEL011153 reverse	TGCTCGGCTTTGAAGAACCC
AAEL012262 forward	AAACAGCCATCAAGCAATGTG
AAEL012262 reverse	CGCTACGCAATGAAGGGAGA
AAEL013571 forward	TCATCCTGGTAATTGTCACTCAA
AAEL013571 reverse	GGGTTTAATAGACGATGGAAACGG
AAEL018211 intron 1 forward	CCTTCACGCAATGTCTGGGA
AAEL018211 intron 1 reverse	CGGTCCAGTTCCCTAATTGAA
AAEL018211 intron 2 forward	CCTTCACGCAATGTCTGGGA
AAEL018211 intron 2 reverse	TTGTCAAAGACTGGCTAGAGTGA
AAEL018350 forward	ACTCAAAAGAAATTAGTAATAAGGA
AAEL018350 reverse	CACTTTCTTTGCTTGTTCCC

C. capitata

LOC101448609 forward	TACTATTGACTACGGGTGAAGC
LOC101448609 reverse	TCGAAAATGCGTCGCTGTTG
LOC101449153 intron 1 forward	GCTTCGCCTGCAGGTAGTC
LOC101449153 intron 1 reverse	CATGGGTTTGGCATGTGACG
LOC101449153 intron 2 forward	TCAATGTTAGCTCAATATTTGCCAT
LOC101449153 intron 2 reverse	GACTGAAACGAAGGCGCAAG
LOC101449153 intron 3 forward	AATTGTCGATTGCATTTTGCTCA
LOC101449153 intron 3 reverse	CTCGAGGGCATGGTTACACA
LOC101449503 forward	TGCCAATGTCCATGTAGCAC
LOC101449503 reverse	TTTATTCGAGTGGTACGTCAGC
LOC101450641 forward	TGGTAGGTAGTTAAGTTATTTGACA
LOC101450641 reverse	AGGCTTTTGAAGCTTTTCTCA
LOC101452861 forward	TTCTGGTGGCATACAGGTCG
LOC101452861 reverse	CGTACTGTGCAACTGGAGGT
LOC101452984 forward	ACTTAATAATCACACAATGGCCGA
LOC101452984 reverse	TCTCATCCAATTCGGCAGGC
LOC101457260 forward	TCAGTATCGGCGGCATCAGT
LOC101457260 reverse	TGGAAACCATCGTCTCTATGCT
LOC101459514 intron 1 forward	GCCAAGAGTCGTTGTGGTGT
LOC101459514 intron 1 reverse	TCGCTTTGTTTTCATCCGTCAATTA
LOC101459514 intron 2 forward	AATTGGCGATCCTGCTCAC
LOC101459514 intron 2 reverse	TTTAGAGGTAGACCAGTTATTTCAA
LOC101461218 forward	AACAACTTCGAAAACCTGTAAGCG
LOC101461218 reverse	CTAAGCGAATCCTCGGGTCC

<u>Primers for amplifying other splice forms</u>

<u>Ae. aegypti</u>

AAEL000028 forward	TTTGGTTGTCCAGGTCCCAC
AAEL000028 reverse A	TGTCATTATTTACAAGGTTTGCTGT
AAEL000028 reverse B	TACTTCCGAGAATGCGAGCC
AAEL001898 forward	GACTGGATGCCACTGTCCTG
AAEL001898 reverse	CTAGAGCGTATCCTGCCAGC
AAEL002083 forward A	CAGATTCGTCCTGATCGCCA
AAEL002083 forward B	GTTTTCCCACGTAGCTTCCG
AAEL002083 reverse	TGGCGTTGTCCATGTCTGTT
AAEL008110 forward	AAGGTCTTTCAGCCTCTGGC
AAEL008110 reverse	ACGAATGCGCCGAAATGAAG
AAEL008428 forward	TGCCTTGTCAAGAAAATCCCA
AAEL008428 reverse	TCGTTATCGCTGAGGCATCC
AAEL011153 forward	TCTGCCTTGCCAGTGATTGA
AAEL011153 reverse	CTTGGAAAAGAACCCTCACGC
AAEL018211 forward	CCATCTTTCGACGCGTGTTC
AAEL018211 reverse	TTCAAAGCGCACGTTGATCC
AAEL018350 forward	ACAAGCTGCTCCAACTCCTC
AAEL018350 reverse	TCGGACGGTGATGGTTTTCC

<u>C. capitata</u>

LOC101448609 forward	TAGGCCATGCACAAGTTGGT
LOC101448609 reverse	TCGAAAATGCGTCGCTGTTG
LOC101449153 forward	TTTGCTCACTCTTCTCGCGT
LOC101449153 reverse	CTCGAGGGCATGGTTACACA
LOC101450641 forward	CAAGCCTTTCGCCTTCGTTC
LOC101450641 reverse	TGCCAACTCGGTGTTGTCTG
LOC101452861 forward	CTGTTGCTGTTGGAGAAGGGT
LOC101452861 reverse	TCTGCCATCACATCAGCAGTT
LOC101452984 forward A	GGCAGTAAACAATCGACGAC
LOC101452984 forward B	CCGAACTCGTTGCTTTCCG
LOC101452984 reverse	GCGAAATCATTGGGACCCTC
LOC101457260 forward	CTGTAGACGCACGGTTTCCA
LOC101457260 reverse	AACGTGTCGTAGATGAGGACG
LOC101459514 forward	ATGATGTTACCAAATGGCCAAATA
LOC101459514 reverse	CATTGCACCGATTTATTGGTTTC
LOC101461218 forward	TCGCCTGTACATTCTTGCGT
LOC101461218 reverse	CATCCGATAAAACGCCAGCC

qRT-PCR primers

Primers for amplifying predicted testis-specific splice form

Ae. aegypti

AAEL000028 forward	CAGTTGGGAGGCTTCGGTAG
AAEL000028 reverse	CCGTTTTAGTTGGAAAATTGTTTGC
AAEL001898 forward	TTCAGTGTGCTTTGGAGGTGT
AAEL001898 reverse	CCCAGTACATTTAAAAAGCATGAAG
AAEL008110 intron 1 forward	GGCAATCAAACCCTTGATTCTGT
AAEL008110 intron 1 reverse	TGGCCGATGTCGAGTAAACC
AAEL011153 forward	TCTGCCTTGCCAGTGATTGA
AAEL011153 reverse	TGCTCGGCTTTGAAGAACCC
AAEL012262 forward	TCGCCACTACTCGTGCTATTC
AAEL012262 reverse	TCACATTGCTTGATGGCTGT
AAEL018211 intron 1 forward	ATCAAAGCCGCATAGCTTGG
AAEL018211 intron 1 reverse	CGGTCCAGTTCCCTAATTGAA
AAEL018211 intron 2 forward	GCTCACCTCGCTTCAGGATT
AAEL018211 intron 2 reverse	TTGTCAAAGACTGGCTAGAGTGA
AAEL018350 forward A	CCCAAAACCAATGAGTAATAAGGA
AAEL018350 forward B	ACTCAAAAGAAATTAGTAATAAGGA
AAEL018350 reverse	GTAGGTGTCTTAGCAGTGAC

C. capitata

LOC101449153 intron 1 forward	GCTTCGCCTGCAGGTAGTC
LOC101449153 intron 1 reverse	CGCCACGTTCCAATAGTTGT
LOC101449153 intron 3 forward	AATTGTCGATTGCATTTTGCTCA
LOC101449153 intron 3 reverse	AGAGAATGCGTGAACAACAAGAT
LOC101449503 forward	TCTTCGACGGTCCAGGACTA
LOC101449503 reverse	TTTATTCGAGTGGTACGTCAGC
LOC101450641 forward	TGGTAGGTAGTTAAGTTATTTGACA
LOC101450641 reverse	AGGCTTTTGAAGCTTTTCTCA
LOC101452861 forward	TTCTGGTGGCATACAGGTCG
LOC101452861 reverse	AGCTGTCGAATGAAGTGCGT
LOC101457260 forward	CCTTTCGGCTGGCTTGGATA
LOC101457260 reverse	TGGAAACCATCGTCTCTATGCT
LOC101459514 intron 1 forward	TCTTCTGGAGAAGCAACTGGG
LOC101459514 intron 1 reverse	TCGCTTTGTTTTCATCCGTCAATTA
LOC101459514 intron 2 forward	TGAGCAGCGATTTCTTCTGTT
LOC101459514 intron 2 reverse	TTTAGAGGTAGACCAGTTATTTCAA

Primers for amplifying other splice forms

Ae. aegypti

AAEL000028 forward	CAGTTGGGAGGCTTCGGTAG
AAEL000028 reverse A	TGTCATTATTTACAAGGTTTGCTGT
AAEL000028 reverse B	AAAGCCACTGGTTTGCTGTG
AAEL001898 forward	GCTGTCTATTGTTGGAGTCCTTTT
AAEL001898 reverse	ACCCAACAGCCATCGATACG
AAEL008110 intron 1 forward	TTCCGACTTTGCCTGGAACA
AAEL008110 intron 1 reverse A	ATGCAAGTTTCGTCCGTCCT
AAEL008110 intron 1 reverse B	CTCGGATGCTGAGGTCCTG
AAEL011153 forward	TCTGCCTTGCCAGTGATTGA
AAEL011153 reverse	CTTGGAAAAGAACCCTCACGC
AAEL01226 forward	TCGCCACTACTCGTGCTATTC
AAEL01226 reverse A	GCGTTTGCGACCTGTTTGT
AAEL01226 reverse B	GGGATGCAGGATATCTGAAACTG
AAEL018211 intron 1 forward A	TTTGAGGGCATTTCCTCGCT
AAEL018211 intron 1 reverse A	TCCAAGTCCGCAAGACAAGG
AAEL018211 intron 1 forward B	AGGGCATTTCATTTGGAGCA
AAEL018211 intron 1 reverse B	CGCACGTTGATCCCACAGAT
AAEL018211 intron 2 forward	GCAGCCTCTCACAAATGTCG
AAEL018211 intron 2 reverse A	CCTGAAGCGAGGAAATGCCC
AAEL018211 intron 2 reverse B	GCTCCAAATGAAATGCCCTCA
AAEL018350 forward A	CCCAAAACCAATGAGTAATAAGGA
AAEL018350 forward B	ACTAGCAGTATAGGTAATAAGGAGA
AAEL018350 reverse	GTAGGTGTCTTAGCAGTGAC

C. capitata

LOC101449153 forward	TTTGCTCACTCTTCTCGCGT
LOC101449153 reverse	TGGCAAATATTGAGTACCAACTGA
LOC101449503 forward	CGTAGGTCGAATAAATCTCAACATC
LOC101449503 reverse	TGTTTTCGTATTGATTTTCCCTGG
LOC101450641 forward	ATAACAAGAGCACCCGCATC
LOC101450641 reverse A	ATTTGGAAAAACTTAACTACCTACC
LOC101450641 reverse B	CGAGGACCCACACGAGAAAG
LOC101452861 forward	TTCTCTGCTGTGCCTCATCG
LOC101452861 reverse	TCTGCCATCACATCAGCAGTT
LOC101457260 forward	GTGTATCTGTAGACGCACGGT
LOC101457260 reverse	ACCTTTGAAGTGAAAATTTTGGATG
LOC101459514 forward A*	ACTGGTCTACCTCTAAATGAACA
LOC101459514 reverse A*	TGAGCAATAGCCAGTTGTCC
LOC101459514 forward B	TTGCAGAATAACTGGTCTACCTCT
LOC101459514 reverse B	TAGGGCAAACTACTGCACCG

*Also targets predicted testis-specific splice forms
